# A novel approach to incorporate frontier areas into urban–rural geographic classifications: Integrated Metropolitan‐to‐Frontier Area Codes

**DOI:** 10.1111/jrh.70102

**Published:** 2025-11-29

**Authors:** Brody J. Gibson, Elizabeth Dobis, John Cromartie, Laurie Grieshober, Cornelia Ulrich, Tracy Onega, Jennifer Doherty

**Affiliations:** ^1^ Huntsman Cancer Institute University of Utah Salt Lake City Utah USA; ^2^ Department of Population Health Sciences, Spencer Fox Eccles School of Medicine University of Utah Salt Lake City Utah USA; ^3^ Rural Economy Branch, Resource and Rural Economics Division, US Department of Agriculture Economic Research Service Kansas City Missouri USA

**Keywords:** distance, frontier, health access, remoteness, rural

## Abstract

**Purpose:**

To develop an urban–rural–frontier classification that integrates urbanicity and geographic remoteness and captures nuances in population and land area distributions invisible in traditional coding schemes, thereby providing a framework to measure health outcomes and access to care across the full urban‐to‐frontier continuum.

**Methods:**

We created tract‐level Integrated Metropolitan‐to‐Frontier Area Codes (tIMFAC) by combining the US Department of Agriculture's Economic Research Service's Frontier and Remote Area (FAR) codes with Rural–Urban Commuting Area (RUCA) codes, classifying tracts as metropolitan, micropolitan, frontier–micropolitan, small town/rural, and frontier–small town/rural. We compared population and land area distributions and median distances to health care facility types by RUCA, FAR, and tIMFAC, and summarized distances to health care facilities across tIMFAC by Census regions.

**Findings:**

tIMFAC metropolitan, micropolitan, and small town/rural categories had higher population densities (312, 74, and 27/m^2^, respectively) than their RUCA counterparts (304, 54, and 11/m^2^, respectively). Densities were much lower in tIMFAC frontier–micropolitan and frontier–small town/rural areas (23 and 4/m^2^, respectively) than micropolitan and small town/rural. Three patterns emerged for travel distances across tIMFAC: (1) steadily increasing distances from metropolitan to frontier–small town/rural areas (e.g., medical‐surgical intensive care units (ICUs)); (2) similar distances within frontier–micropolitan and micropolitan, and within frontier–small town/rural and small town/rural, respectively (e.g., obstetrics); and (3) longer distances for frontier areas regardless of urbanicity (e.g., pediatric ICUs and designated trauma centers).

**Conclusion:**

tIMFAC provides a policy‐relevant approach to identifying health differences across the urban‐to‐frontier continuum, supporting efforts to better understand and address unique rural and frontier health challenges.

## THE NEED FOR A MORE NUANCED UNDERSTANDING OF RURALITY USING MEASURES OF URBANICITY AND GEOGRAPHIC REMOTENESS

Geographic remoteness describes the degree to which a community is physically isolated from larger population centers and the infrastructure, services, and opportunities they provide. In health research, this concept is important for understanding access to care, health outcomes, and service availability, particularly in rural and frontier areas.^1^ Residents of frontier areas in the United States (US) face unique challenges in obtaining essential resources and basic health services, largely due to extreme remoteness.^2^ Longer travel times and distances to care have been linked with delayed treatment, lower preventive service use, and poorer health outcomes in rural populations.^3^, ^4^, ^5^, ^6^ Even travel times of 30 min can reduce utilization, while those exceeding 60 min for specialty care can hinder effective management of chronic conditions and maternal health.^3^

The US Department of Agriculture's Economic Research Service (USDA‐ERS) created the 2010 Frontier and Remote (FAR) area codes, which classify frontier areas at the ½ × ½ kilometer grid level using travel time to the nearest urban area. FAR codes are organized into four nested levels, with Level 1 representing areas that are ≥60 min from an urban area of ≥50,000 people and subsequent levels (2–4) denoting increasing degrees of remoteness.^2^ According to FAR Level 1, a little over half of the US land area was considered frontier, with 12.2 million residents living in those areas in 2010.^7^

Most studies of geographic health differences rely on traditional urban–rural classifications.^8^, ^9^ These classifications typically begin with Census Bureau definitions, which identify urban areas as either urbanized areas (UA, population ≥50,000 residents) or urban clusters (UC, population between 2,500 and 50,000 residents), with all remaining areas defined as rural.^9^ The Office of Management and Budget (OMB) expanded on these definitions to delineate core‐based statistical areas (CBSAs),^8^ classifying counties into metropolitan or micropolitan statistical areas based on the population size of urban areas and commuting ties.^10^ At the finer census tract scale, the USDA‐ERS developed Rural–Urban Commuting Area (RUCA) codes using a similar framework.^11^

CBSA‐based classifications largely function as indicators of urbanicity, reflecting characteristics commonly associated with urban areas such as high population density, transportation networks, and access to services.^12^, ^13^ However, they do not account for geographic remoteness. Areas with comparable urbanicity can differ substantially in geographic remoteness, limiting the utility of existing classifications for studying access to care. Moreover, common measures of rurality often oversimplify rural areas by defining them primarily as nonurban, masking important variations in population and regional characteristics.^9^

To address these gaps, we present the census tract‐level Integrated Metropolitan‐to‐Frontier Area Codes (tIMFAC), a novel classification that combines the USDA‐ERS RUCA urbanicity measures with USDA‐ERS FAR travel time measures of geographic remoteness. In this paper, we describe the development of tIMFAC and characterize US population and land area distributions across tIMFAC categories. Additionally, we calculate median travel distances to various health care facilities across tIMFAC categories to explore how urbanicity and remoteness together shape access to care.

## METHODS

### Conceptual framework for an integrated urbanicity and remoteness classification

**FIGURE 1:**
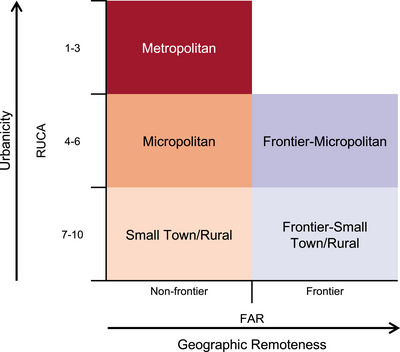
Conceptual framework for classifying US areas along two spatial dimensions: urbanicity and geographic remoteness. Integrating these two dimensions enables more nuanced comparisons of potential access to essential resources and services because it reflects both how areas are structured and connected (urbanicity) and how isolated they are from larger population centers (remoteness).

The framework used in this study conceptualizes US areas along two spatial dimensions: urbanicity and geographic remoteness (Figure 1). Urbanicity is measured using the publicly‐available 2010 census tract‐level RUCA codes developed by the USDA‐ERS, which delineate areas based on population density, urbanization, and commuting patterns. RUCA identifies three core urban area types by size: metropolitan cores (UA population ≥50,000), micropolitan cores (UC 10,000–49,999), and small‐town cores (UC 2500–9999), as well as rural areas with no linkage to core areas. Lower RUCA codes correspond to larger, more urbanized areas, whereas higher codes reflect smaller, more rural areas.

Geographic remoteness is measured using unpublished 2010 census tract‐level FAR level 1 codes provided by the USDA‐ERS, which classifies areas by travel time to the edge of the nearest urban area of a given size. Under FAR level 1, “frontier” areas are those located 60 min or more by vehicle from the boundary of the nearest urban area with a population of at least 50,000 (Table S1 provides detailed category definitions for each coding scheme).

By integrating these two dimensions, the tIMFAC classification establishes five mutually‐exclusive categories: metropolitan, micropolitan, frontier–micropolitan, small town/rural, and frontier–small town/rural. This framework enables more nuanced comparisons of potential access to essential resources and services because it reflects both how areas are structured and connected (urbanicity) and how isolated they are from larger population centers (remoteness).

### Creating the Integrated Metropolitan‐to‐Frontier Area Codes

We first grouped US census tracts by their assigned primary RUCA code categories: metropolitan (1–3), micropolitan (4–6), and small town/rural (7–10). We cross‐classified these by two categories defined by FAR codes: frontier (FAR level 1, FAR = 1), and non‐frontier (no FAR code, FAR = 0). This resulted in six mutually exclusive categories: metropolitan (RUCA 1–3 and FAR = 0), micropolitan (RUCA 4–6 and FAR = 0), small town/rural (RUCA 7–10 and FAR = 0), frontier‐metropolitan (RUCA 1–3 and FAR = 1), frontier–micropolitan (RUCA 4–6 and FAR = 1), and frontier–small town/rural (RUCA 7–10 and FAR = 1). We excluded 278 census tracts that did not have a RUCA code assignment, leaving 72,779 census tracts in our final dataset.

A small number of census tracts were identified as frontier‐metropolitan (*n* = 36; 95,256 people). The small population size of this group (0.03% of the total US population) presents technical challenges for analysis, including unstable estimates, limited power for subgroup comparisons, and increased disclosure risk in small‐area data. None of the 36 tracts were RUCA code 1, meaning they were not a census tract containing a UA of ≥50,000 people—but were instead functionally tied to the UA by having commuting flows of ≥30% (RUCA 2) or 10% to <30% (RUCA 3). As all tracts met the FAR definition of frontier, they are also located at least 60 min from a UA of ≥50,000 people. In determining an appropriate reclassification, it was important to preserve the commuting‐based logic of RUCA while aligning with the geographic remoteness captured by FAR. Micropolitan tracts (RUCA 4–6) are defined as having an UC of 10,000–49,999 (RUCA 4) or being functionally tied to micropolitan areas by having commuting flows of ≥30% (RUCA 5) or 10% to <30% (RUCA 6). In contrast, small town/rural areas (RUCA 7–10) are tied to smaller UCs of 2,500 to 9,999 or lack urban connectivity altogether. We therefore reclassified the frontier‐metropolitan tracts as frontier–micropolitan given their similarities with respect to commuting flows.

**FIGURE 2:**
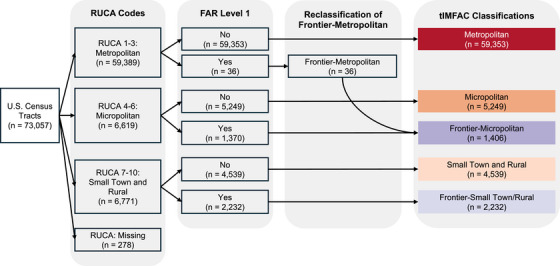
Diagram illustrating the combined classification of US census tracts using the USDA‐ERS Rural–Urban Commuting Area (RUCA) and Frontier and Remote (FAR) Area codes to develop the tract‐level Integrated Metropolitan‐to‐Frontier Area Codes (tIMFAC) scheme to assign tracts as metropolitan, micropolitan, small town/rural, frontier–micropolitan, and frontier–small town/rural.

**FIGURE 3:**
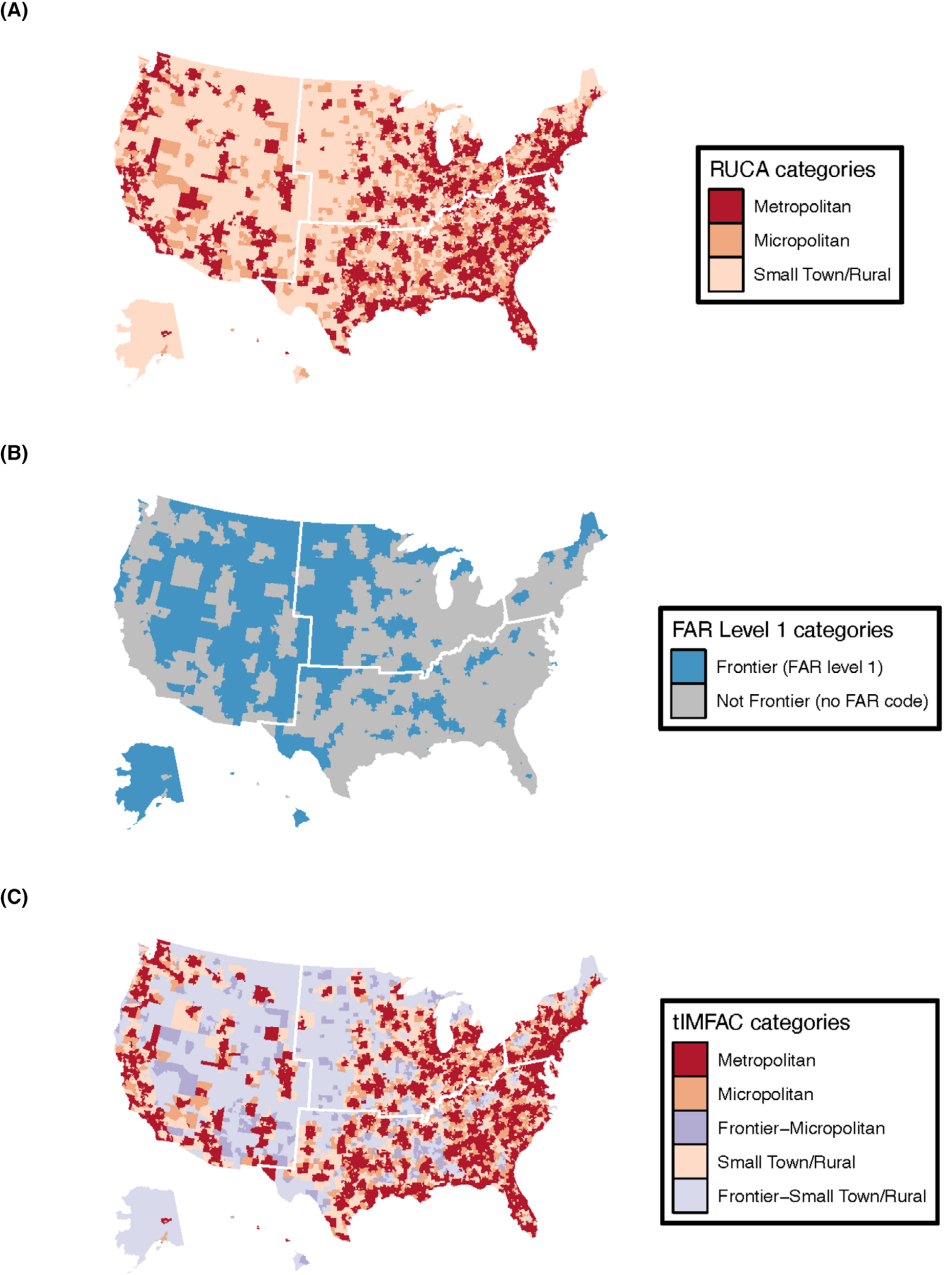
Maps of the tract‐level (A) RUCA codes, (B) FAR level 1 codes, and (C) tIMFAC. *Note*: White borders indicate US Census Region borders. Regions are defined as follows: Northeast (Connecticut, Maine, Massachusetts, New Hampshire, New Jersey, New York, Pennsylvania, Rhode Island, Vermont); Midwest (Illinois, Indiana, Iowa, Kansas, Michigan, Minnesota, Missouri, Nebraska, North Dakota, Ohio, South Dakota, Wisconsin); South (Alabama, Arkansas, Delaware, District of Columbia, Florida, Georgia, Kentucky, Louisiana, Maryland, Mississippi, North Carolina, Oklahoma, South Carolina, Tennessee, Texas, Virginia, West Virginia); and West (Alaska, Arizona, California, Colorado, Hawaii, Idaho, Montana, Nevada, New Mexico, Oregon, Utah, Wyoming, Washington). All maps were created in R using the “ggplot2” package.

Figure 2 outlines the steps used to create the tIMFAC classifications, illustrating how RUCA and FAR codes were combined to define the five categories. To provide spatial context, Figure 3 presents maps of census tract‐level RUCA codes, FAR level 1 designations, and the resulting tIMFAC categories. We also assessed the distribution of FAR level 1–4 codes across all of the frontier designations (Table S2).

To support analyses at multiple geographic scales, we also developed a county‐level version of the IMFAC classification with comparable categories. The availability of IMFAC codes at both census tract and county levels enables researchers and policymakers to analyze and compare data across different spatial units. Furthermore, it offers additional flexibility when analyzing health data under strict county‐level restrictions intended to protect patient privacy. Details on the development of the county‐level IMFAC (cIMFAC) classification are provided in Section B of the Supplementary Materials.

### Population, land area, and distance to health care facilities by RUCA, FAR, and tIMFAC

Census tract‐level population data were downloaded from the 2010 Census of Population and Housing (Summary File 1).^14^ We used 2010 population data to ensure consistency with the RUCA and FAR codes, which were both also derived from the 2010 Census. We obtained land area and boundary line data at the tract level from the 2010 TIGER/Line Shapefiles.^15^ The distribution of US census tracts, population, land area (per square mile, m^2^), and population density (persons/m^2^) were summarized across RUCA categories, FAR level 1, and tIMFAC census tract classifications.

Census tract‐level distance measures to the nearest hospital and other types of health care facilities in 2010 were downloaded from the Agency for Healthcare Research and Quality's (AHRQ's) Social Determinants of Health (SDOH) database.^16^ The AHRQ calculated distances to hospitals and other types of health care facilities using data from the Centers for Medicare and Medicaid Services (CMS) Provider of Services (POS) files. Distance was measured in miles from population‐weighted tract centroids to the nearest health clinic (Federal Qualified Health Centers and Rural Health Clinics), as well as to the nearest hospital with an emergency department (ED), medical‐surgical intensive care unit (ICU), designated trauma center, pediatric ICU, or inpatient care for alcohol and drug abuse.^16^ Median distances to each type of health care facility were calculated for all RUCA, FAR level 1, and tIMFAC categories, for the US overall, and for tIMFAC categories by Census regions.^17^

## RESULTS

### Comparisons between RUCA, FAR, and tIMFAC classifications

**FIGURE 4:**
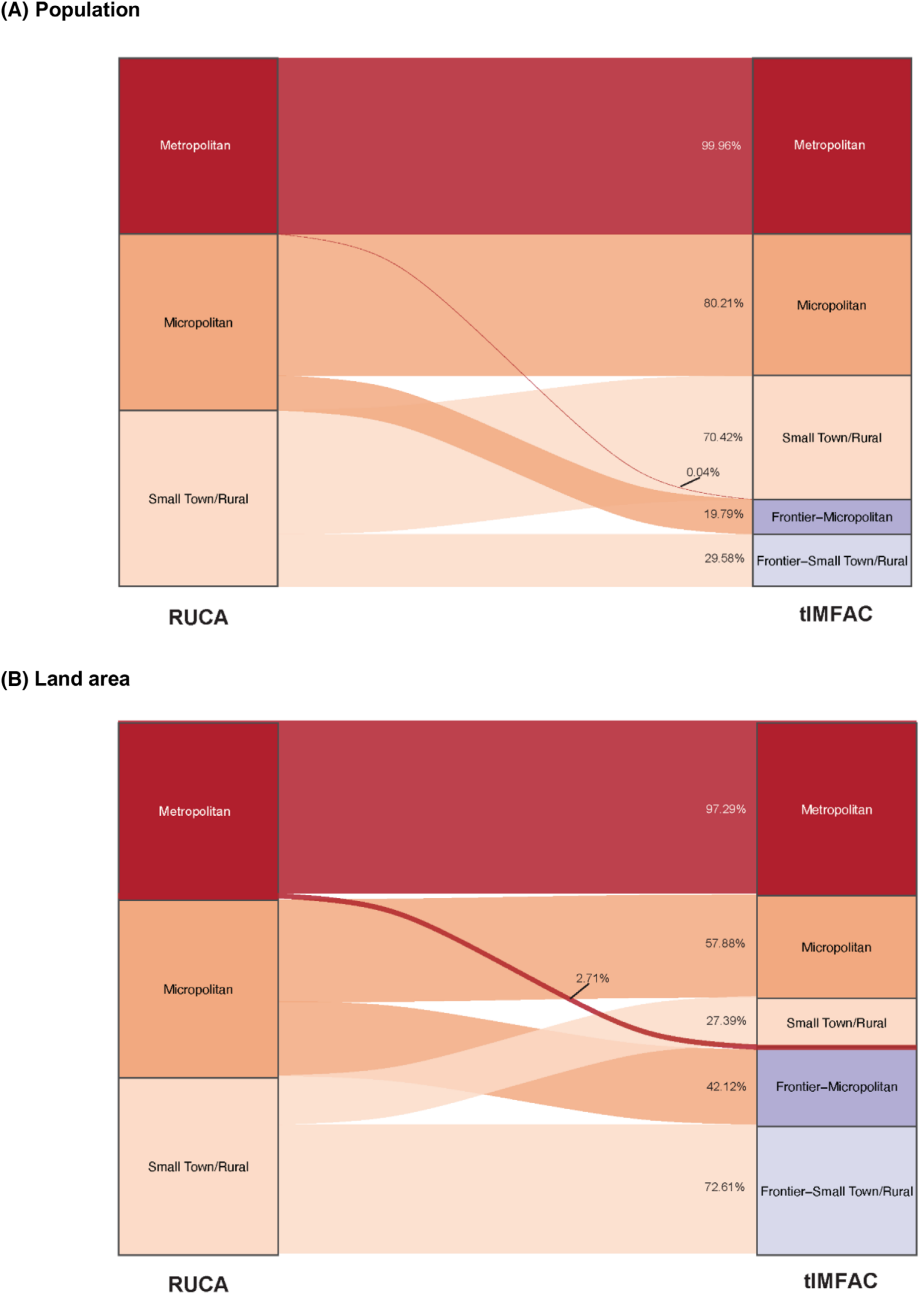
The population (A) and land area (B) percentages of each RUCA category reclassified into each tIMFAC category. *Note*: Flow widths represent the percentage of each RUCA category reclassified into each tIMFAC category.

Table 1 shows the US population, land area, population density, and census tracts in 2010 classified by RUCA, FAR, and tIMFAC. RUCA and tIMFAC metropolitan classifications both accounted for 84% of the population, but populations in tIMFAC micropolitan (7.2%) and small town/rural (5.3%) were reduced due to some of the population being classified as frontier–micropolitan (1.8%) or frontier–small town/rural (2.2%). In terms of land area, tIMFAC assigned 23% to metropolitan, 8.5% to micropolitan, 6.8% to frontier–micropolitan, 17% to small town/rural, and 45% to frontier–small town/rural. The frontier subcategories represented 20% and 30% of RUCA's micropolitan and small town/rural populations, respectively (Figure 4a). Of RUCA micropolitan land area, 42% was frontier–micropolitan in tIMFAC, while 73% of RUCA small town/rural land area was frontier–small town/rural (Figure 4b).

**TABLE 1:** US population and land area classified according to tract‐level RUCA, FAR, and tIMFAC geographic schemes, 2010.

Scheme	Classification	Population (*n* = 308,745,538)	Land area (m^2^)	Population density (persons/m^2^)	Tracts	% Population	% Land area
**RUCA**	**Metropolitan**	257,810,493	847,813	304	59,389	84	24
	**Micropolitan**	27,788,710	518,269	54	6,619	9.0	15
	**Small town/rural**	23,146,335	2,165,720	11	6,771	7.5	61
**FAR** ^a^	**Frontier**	12,443,026	1,813,820	7	3,638	4.0	51
**tIMFAC**	**Metropolitan**	257,715,237	824,846	312	59,353	84	23
	**Micropolitan**	22,288,352	299,979	74	5,249	7.2	8.5
	**Small town/rural**	16,298,923	593,157	27	4,539	5.3	17
	**Frontier–micropolitan**	5,595,614	241,256	23	1,406	1.8	6.8
	**Frontier–small town/rural**	6,847,412	1,572,563	4	2,232	2.2	45
	***(Frontier total)*** ^b^	*(12,443,026)*	*(1,813,820)*	*(7)*	*(3,638)*	*(4.0)*	*(51)*

*Source*: US Census Bureau, Census of Population and Housing, 2010. Summary File 1. US Census Bureau. Tiger/Line Shapefiles, Census Tracts and Urban Areas, 2010.

Abbreviations: RUCA: Rural–Urban Commuting Area; FAR: Frontier and Remote; tIMFAC: tract‐level Integrated Metropolitan‐to‐Frontier Area Codes.

^a^ FAR frontier is defined as FAR level 1.

^b^ tIMFAC frontier total includes frontier–micropolitan and frontier–small town/rural combined. These two tIMFAC frontier categories combined comprise all tracts defined by FAR level 1 as frontier.

Population densities were consistently higher for tIMFAC metropolitan, micropolitan, and small town/rural categories (312, 74, and 27 persons/mile^2^, respectively) compared with their RUCA counterparts (304, 54, and 11, respectively), reflecting the removal of large, sparsely populated frontier areas from these categories in tIMFAC. While frontier census tracts overall accounted for 51% of the land area with a population density of 7 persons/m^2^, there was considerable variation between the frontier subcategories. Frontier–micropolitan tracts accounted for only 6.8% of the land area, with a population density of 23 persons/m^2^, and frontier–small town/rural accounted for 45% of the land area, with a population density of 4 persons/m^2^.

The distribution of the nested FAR levels (1–4) in the frontier subcategories (Table S2) showed that 41% of frontier–small town/rural census tracts were FAR level 4 (the most geographically remote category), while only 3.1% of frontier–micropolitan areas were in that category.

### Median travel distances to health care facilities

**FIGURE 5:**
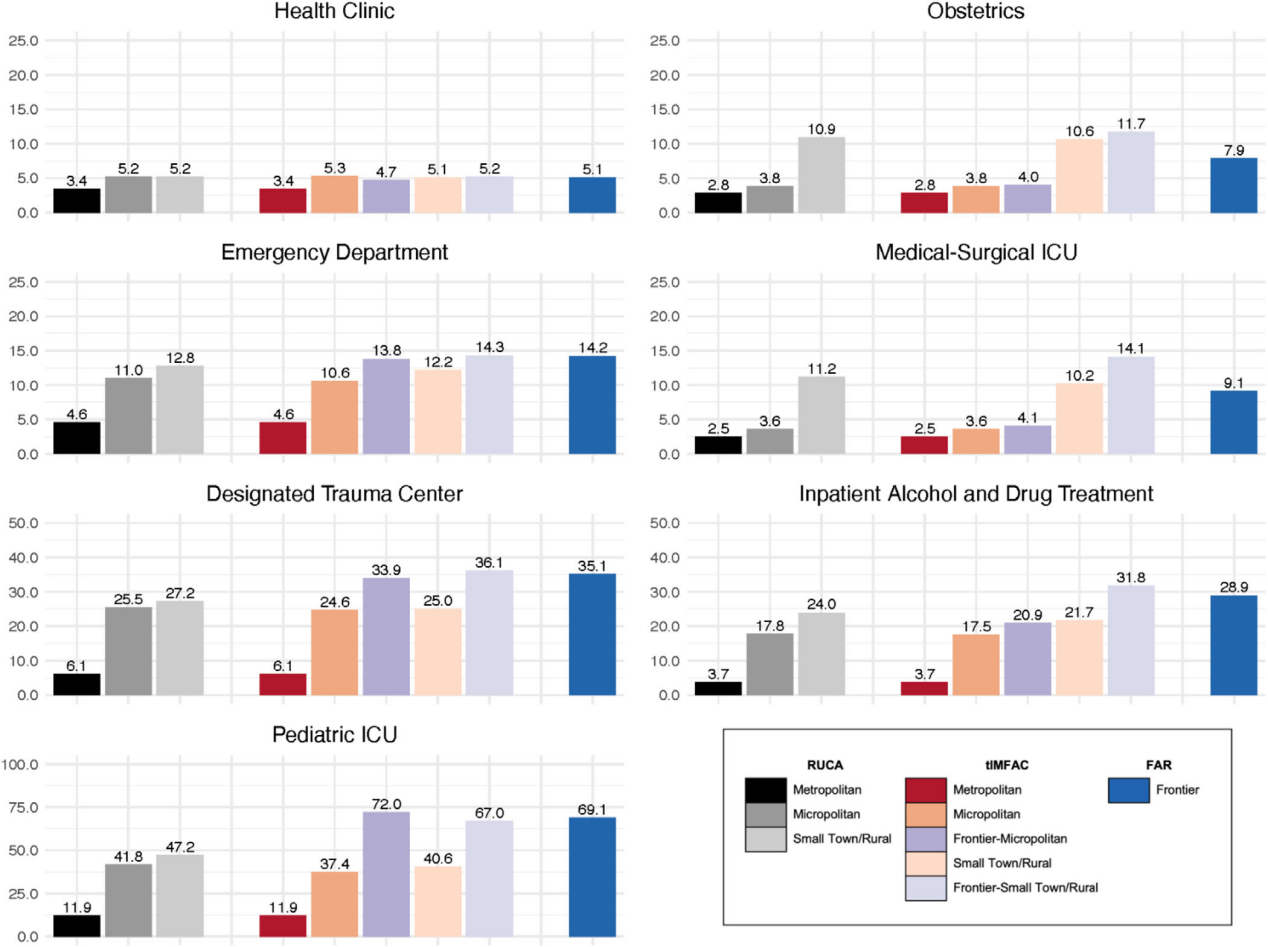
Bar plots of the median distance (in miles) to health facilities by RUCA, tIMFAC, and FAR level 1 codes for the overall United States in 2010 *Source*: AHRQ Social Determinants of Health Database, 2010 Census Tract Data *Note*: Of the 72,779 census tracts assigned a tIMFAC code, 72,633 had median distance to health facilities measures in 2010. The 146 tracts with missing distance measures were excluded.

Median travel distances to health care facilities in 2010 by RUCA, FAR, and tIMFAC revealed distinct patterns across these classifications (Figure 5). For RUCA, there was generally a consistent increase in median distance to health care facilities from metropolitan to micropolitan to small town/rural. FAR‐defined frontier areas demonstrated longer median travel distances than RUCA‐defined small town/rural areas for most facility types, including emergency department, pediatric ICU, designated trauma center, and inpatient alcohol and drug treatment facilities. tIMFAC‐defined micropolitan and small town/rural categories generally exhibited median distances comparable to those defined by RUCA. However, when compared to FAR‐defined frontier areas, tIMFAC‐defined frontier–small town/rural areas had longer median travel distances, while tIMFAC‐defined frontier–micropolitan areas had shorter distances (with the exception of pediatric ICUs).

Across tIMFAC categories, three overarching patterns in travel burden emerged, each reflecting distinct relationships. First, for medical‐surgical ICUs, travel distances increased steadily from metropolitan to frontier–small town/rural areas, reflecting a clear gradient. Second, for obstetrics, median travel distances were similar between frontier and non‐frontier areas within the same urbanicity (e.g. frontier–micropolitan and micropolitan; frontier–small town/rural and small town/rural). These findings suggest that for certain types of care, frontier designation does not always exacerbate travel burden within similar urbanicity levels. Third, for specialized services such as pediatric ICUs and designated trauma centers, both frontier–micropolitan and frontier–small town/rural consistently exhibited longer travel distances than their non‐frontier counterparts. This pattern highlights compounded challenges faced by frontier populations in accessing specialized care regardless of urbanicity.

**FIGURE 6:**
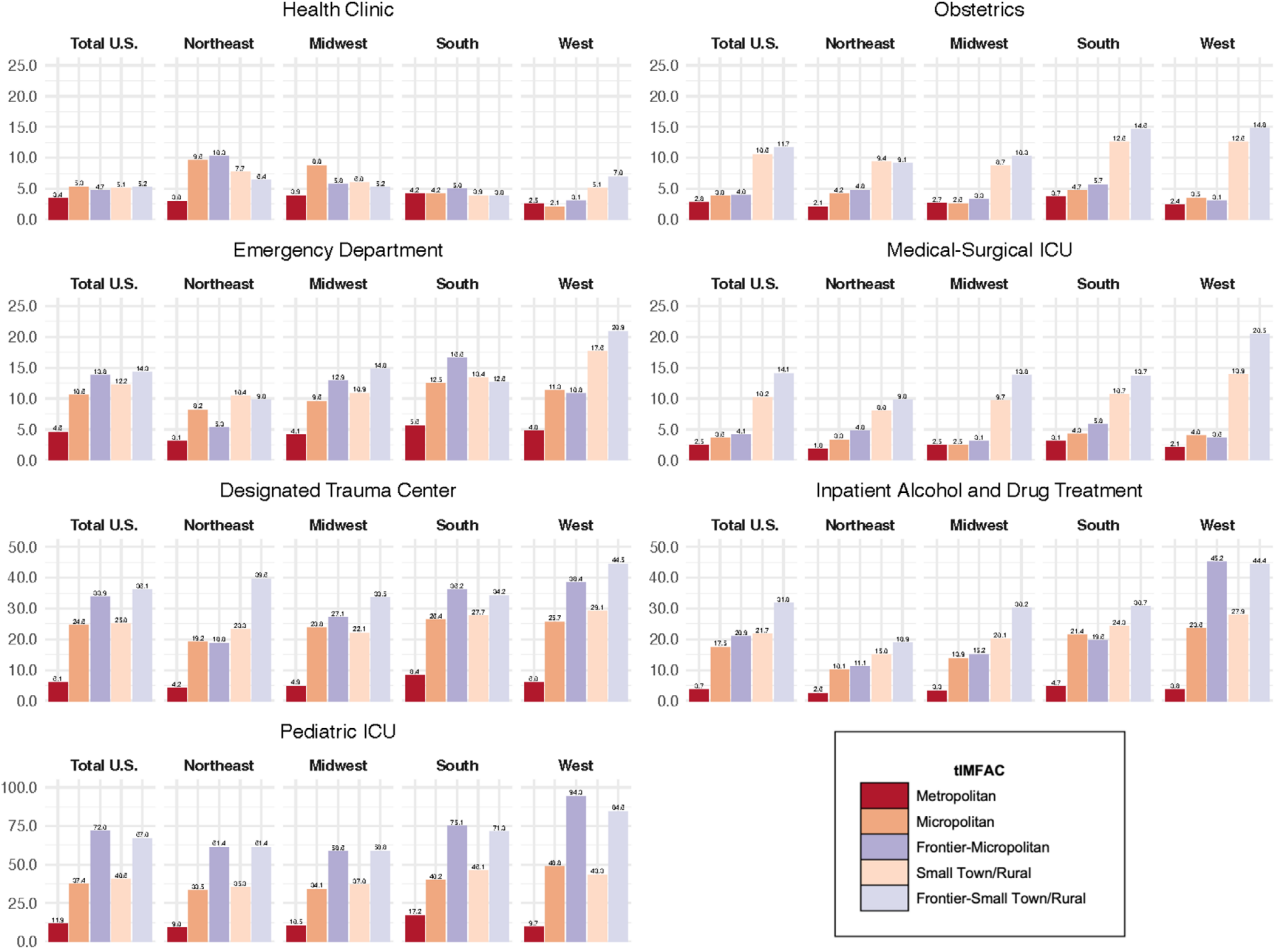
Bar plots of the median distance (in miles) to health facilities by tIMFAC for the United States overall and by US Census Bureau‐defined regions in 2010. *Source*: AHRQ Social Determinants of Health Database, 2010 Census Tract Data *Note*: Of the 72,779 census tracts assigned a IMFAC code, 72,633 had median distance to health facilities measures in 2010 Regions are defined as follows: Northeast (Connecticut, Maine, Massachusetts, New Hampshire, New Jersey, New York, Pennsylvania, Rhode Island, Vermont); Midwest (Illinois, Indiana, Iowa, Kansas, Michigan, Minnesota, Missouri, Nebraska, North Dakota, Ohio, South Dakota, Wisconsin)**;** South (Alabama, Arkansas, Delaware, District of Columbia, Florida, Georgia, Kentucky, Louisiana, Maryland, Mississippi, North Carolina, Oklahoma, South Carolina, Tennessee, Texas, Virginia, West Virginia); and West (Alaska, Arizona, California, Colorado, Hawaii, Idaho, Montana, Nevada, New Mexico, Oregon, Utah, Wyoming, Washington).

Regional patterns of median distances across tIMFAC categories were generally similar to national patterns (Figure 6). For all services except for health clinics, the West region had longer median travel distances than other regions, particularly for frontier–micropolitan and frontier–small town/rural areas. Patterns of distance to health clinics varied considerably by region. Unlike other regions, the Northeast had longer median travel distances to health clinics across all tIMFAC categories compared to the total US, with the exception of metropolitan areas. We performed sensitivity analyses to assess the influence of large states, including Alaska, Montana, and Texas; excluding each of these from their respective regions did not change interpretations (Figures S1–S3).

## DISCUSSION

The tIMFAC classification framework addresses key limitations in existing geographic classifications by integrating urbanicity and geographic remoteness measures into a single classification, and splitting micropolitan and small town/rural areas into frontier and non‐frontier subcategories. This simultaneously allows for the assessment of frontier populations by their degree of urbanicity.

The tIMFAC classification reveals nuances in population and land area distribution invisible in RUCA and FAR codes. For example, approximately 40% of micropolitan land and one‐fifth of its population were reclassified as frontier–micropolitan, while about 70% of small town/rural land and one‐third of its population were reclassified as frontier–small town/rural. Frontier–small town/rural areas encompass the largest proportion of land but have the lowest population densities, reflecting the extreme sparsity and isolation of these areas. In contrast, frontier–micropolitan areas, while still remote, have a higher population density due to their population size and economic integration with urban centers.

Differences in population density are mirrored in the median travel distance to health care facilities. Consistent with prior reports,^18^ tIMFAC‐defined metropolitan areas exhibited the shortest travel distances across all facility types, similar to patterns observed in RUCA‐defined metropolitan areas. However, tIMFAC provides additional granularity by distinguishing frontier and non‐frontier areas within urbanicity levels, revealing meaningful variation in health care access. One prominent pattern was the progressive increase in travel distance observed from metropolitan to frontier–small town/rural areas, particularly for services such as medical‐surgical ICUs. In contrast, certain facility types (e.g., obstetrics services) showed relatively similar travel distances between frontier and non‐frontier areas within the same urbanicity category, suggesting that frontier designation does not uniformly exacerbate access challenges.

An additional pattern emerged for specialized services, including pediatric ICUs and designated trauma centers, where frontier areas consistently faced longer travel distances than non‐frontier counterparts. For pediatric ICUs, distances for frontier areas ranged from approximately 58 miles in the Midwest to 94 miles in the West. While the utilization of these services is often clinically necessary, such distances impose financial and logistical burdens on families and caregivers (e.g., costs for travel and lodging).^19^ These burdens compound existing rural health disparities and may indirectly influence outcomes by delaying follow‐up care or reducing caregiver capacity. Similarly, longer travel times to emergency departments and trauma centers reached 21 miles and 45 miles, respectively, in frontier–small town/rural areas of the West. Prior research shows that longer travel times to emergency care are associated with more complex disease at presentation, higher charges, and longer inpatient stays.^5^ These patterns may extend to preventive services (e.g., lung cancer screening), where travel burden may directly influence utilization.^3^, ^6^ Although some studies have found that distance does not consistently predict health care use,^20^ others report that greater travel distances can reduce utilization and contribute to delays in care.^3^ These findings underscore the value of tIMFAC in capturing meaningful differences in health care access that are obscured in conventional classifications. Collectively, these distinctions enable more precise identification of underserved areas and support targeted policy and resource allocation efforts across the metropolitan‐frontier continuum. While the 2010 IMFAC classifications at the tract and county levels are key for analyzing existing data, updating them with data from each decennial census will be necessary to account for temporal changes in population dynamics, commuting patterns, and urbanization.

The ability to distinguish between frontier–micropolitan and frontier–small town/rural areas provides flexibility for researchers to examine frontier populations either as a unified group or as distinct subcategories. The substantial agreement between tract‐level and county‐level IMFAC (Table B2) supports the reliability of using the IMFAC coding scheme at both geographic levels. This means researchers and policymakers can confidently analyze and compare data across different geographic scales. Moreover, it offers additional flexibility when analyzing health data under strict county‐level restrictions intended to protect patient privacy.

A limitation of this study was the use of the AHRQ median distance to health care facilities calculated from population‐weighted centroid distance data. While these distances are relatively precise proxies of distance traveled by tract residents, they may be less accurate for large or irregularly shaped tracts where the population is unevenly distributed.^21^ It is also important to highlight that a limitation of IMFAC (and other classifications of census tracts or counties) is that the heterogeneity within an area may be obscured by their geographic size.^22^ Despite the limitations of distance to health care facilities measurements, they effectively demonstrate the differences in access to care across the tIMFAC categories.

As Bennett et al. highlighted in *Health Affairs*, broad rural categories often fail to reflect the lived experiences of rural populations, which frequently span multiple administrative jurisdictions and encompass heterogeneous cultural, demographic, and infrastructural characteristics. These limitations can contribute to measurement bias, misinterpretation of research findings, and inequitable resource distribution.^9^ The IMFAC classifications address some of these challenges by incorporating travel time and geographic remoteness into existing CBSA‐based classifications, offering a more refined understanding of access to health care services and population health needs.

## CONCLUSION

The IMFAC scheme offers a refined approach to the classification of urban–rural–frontier areas by integrating urbanicity and geographic remoteness. By combining these measures, IMFAC addresses a key limitation in conventional classification systems–their inability to distinguish geographically remote and sparsely populated frontier areas within broader rural categories. Ultimately, IMFAC provides a more precise and actionable framework for understanding rurality and frontier conditions in the United States, supporting health, policy, and service delivery research across the metropolitan‐to‐frontier continuum.

## CONFLICT OF INTEREST STATEMENT

The authors declare no conflicts of interest.

## Supporting information



Supporting Information

## Data Availability

A copy of the 2010 IMFAC codes with Census tract and county FIPS are available upon request.
