# Supplementary material for: A novel approach to incorporate frontier areas into urban–rural geographic classifications: Integrated Metropolitan‐to‐Frontier Area Codes
Source: J Rural Health. 2025 Nov 29;41(4):e70102. doi: 10.1111/jrh.70102 (PMC12664327; doi:10.1111/jrh.70102)
Supplement: Supplementary file 1 — Supporting Information [file JRH-41-0-s001.docx]

**Supplementary Materials**

Table of Contents

[Section A. Supplementary Tables and Figures 2](#_Toc213679871)

[A1. Tables 2](#_Toc213679872)

[Table S1 2](#_Toc213679873)

[Table S2 3](#_Toc213679874)

[A2. Figures 4](#_Toc213679875)

[Figure S1 4](#_Toc213679876)

[Figure S2 5](#_Toc213679877)

[Figure S3 6](#_Toc213679878)

[Section B. County-level Metropolitan-to-Frontier Area Codes (cIMFAC) 7](#_Toc213679879)

[B1. Methods 7](#_Toc213679880)

[Figure B1 7](#_Toc213679881)

[B2. Summary Tables 8](#_Toc213679882)

[Table B1 8](#_Toc213679883)

[B3. Agreement between cIMFAC and tIMFAC classifications 9](#_Toc213679884)

[Table B2 9](#_Toc213679885)

[B4. Maps 10](#_Toc213679886)

[Figure B2 10](#_Toc213679887)

# **Section A. Supplementary Tables and Figures**

## **A1. Tables**

| Table S1. Category definitions for the census tract-level USDA-ERS 2010 Frontier and Remote (FAR) Area Codes and 2010 Rural-Urban Commuting Area (RUCA) Codes | | |
| --- | --- | --- |
| **Scheme** | **Classification** | **Definition** |
| **FAR Codes^a^** | **Level 1** | Rural areas and urban areas up to 50,000 people that are:   1. ≥ 60 minutes from an urban area of ≥ 50,000 people |
|  | **Level 2** | Rural areas and urban areas up to 25,000 people that are:   1. ≥ 45 minutes from an urban area of 25,000-49,999 people;  2. ≥ 60 minutes or more from an urban area of ≥ 50,000 people |
|  | **Level 3** | Rural areas and urban areas up to 10,000 people that are:   1. ≥ 30 minutes from an urban area of 10,000-24,999 people;  2. ≥ 45 minutes from an urban area of 25,000-49,999 people;  3. ≥ 60 minutes from an urban area of ≥ 50,000 people |
|  | **Level 4** | Rural areas that are:   1. ≥ 15 minutes from an urban area of 2,500-9,999 people;  2. ≥ 30 minutes from an urban area of 10,000-24,999 people;  3. ≥ 45 minutes from an urban area of 25,000-49,999 people;  4. ≥ 60 minutes from an urban area of 50,000 or more people |
| **Primary**  **RUCA Codes^b^** | **1** | Metropolitan area core: primary flow within an urbanized area (UA)^c^ of ≥ 50,000 people |
|  | **2** | Metropolitan area high commuting: primary flow ≥ 30% to a UA |
|  | **3** | Metropolitan area low commuting: primary flow 10% to 30% to a UA |
|  | **4** | Micropolitan area core: primary flow within an Urban Cluster (UC)^d^ of 10,000 to 49,999 (large UC) |
|  | **5** | Micropolitan high commuting: primary flow ≥ 30% to a large UC |
|  | **6** | Micropolitan low commuting: primary flow 10% to 30% to a large UC |
|  | **7** | Small town core: primary flow within an Urban Cluster of 2,500 to 9,999 (small UC) |
|  | **8** | Small town high commuting: primary flow ≥ 30% to a small UC |
|  | **9** | Small town low commuting: primary flow 10% to 30% to a small UC |
|  | **10** | Rural areas: primary flow to a tract outside a UA or UC |
|  | **99** | Not coded: Census tract has zero population and no rural-urban identifier information |
| Sources: U.S. Department of Agriculture - Economic Research Services, Frontier and Remote (FAR) Area Codes, 2010, Census Tract-level U.S. Department of Agriculture - Economic Research Services, Rural-Urban Commuting Area (RUCA) Codes, 2010, Census Tract-level  Notes: ^a^Travel time is measured as the time it takes to travel by car to the edge of a nearby urban area.  ^b^Tracts are defined as cores if > 20% of the tract is in the UA or UC. ^c^Urbanized Areas (UA) are defined as urban areas encompassing ≥ 50,000 people. ^d^Urban Clusters (UC) are defined as urban areas encompassing at least 2,500 and < 50,000 people. | | |

| Table S2. Distribution of nested FAR levels 1-4, primary RUCA codes, and grouped RUCA codes for frontier areas (FAR=1) within RUCA-defined metropolitan, micropolitan, and small town/rural areas, and the final IMFAC-defined frontier-micropolitan and frontier-small town/rural areas | | | | | | | | | | | |
| --- | --- | --- | --- | --- | --- | --- | --- | --- | --- | --- | --- |
| **Scheme** | **Category** | **FAR level 1-defined Frontier Areas** | | | | | | **Final IMFAC-defined Frontier Areas^b^** | | | |
|  |  | **Metropolitan** | | **Micropolitan** | | **Small Town/Rural** | | **Frontier-Micropolitan** | | **Frontier-Small Town/Rural** | |
|  |  | (n = 36) | **%** | (n = 1,370) | % | (n = 2,232) | % | (n = 1,406) | % | (n = 2,232) | % |
| **Nested**  **FAR Levels** | Level 1 | 36 | 100 | 1,370 | 100 | 2,232 | 100 | 1,406 | 100 | 2,232 | 100 |
|  | Level 2 | 30 | 83 | 580 | 42 | 1,821 | 82 | 610 | 43 | 1,821 | 82 |
|  | Level 3 | 28 | 78 | 22 | 1.6 | 1,505 | 67 | 50 | 3.6 | 1,505 | 67 |
|  | Level 4 | 24 | 67 | 20 | 1.5 | 925 | 41 | 44 | 3.1 | 925 | 41 |
| **Primary**  **RUCA** | **1** | 0 | 0 | 0 | 0 | 0 | 0 | 0 | 0 | 0 | 0 |
|  | **2** | 23 | 64 | 0 | 0 | 0 | 0 | 23 | 1.6 | 0 | 0 |
|  | **3** | 13 | 36 | 0 | 0 | 0 | 0 | 13 | 0.9 | 0 | 0 |
|  | **4** | 0 | 0 | 794 | 58 | 0 | 0 | 794 | 56 | 0 | 0 |
|  | **5** | 0 | 0 | 528 | 39 | 0 | 0 | 528 | 38 | 0 | 0 |
|  | **6** | 0 | 0 | 48 | 3.5 | 0 | 0 | 48 | 3.4 | 0 | 0 |
|  | **7** | 0 | 0 | 0 | 0 | 496 | 22 | 0 | 0 | 496 | 22 |
|  | **8** | 0 | 0 | 0 | 0 | 314 | 14 | 0 | 0 | 314 | 14 |
|  | **9** | 0 | 0 | 0 | 0 | 46 | 2.1 | 0 | 0 | 46 | 2.1 |
|  | **10** | 0 | 0 | 0 | 0 | 1,201 | 54 | 0 | 0 | 1,201 | 54 |
| **Grouped RUCA^a^** | Metropolitan | 36 | 100 | 0 | 0 | 0 | 0 | 36 | 2.6 | 0 | 0 |
|  | Micropolitan | 0 | 0 | 1,370 | 100 | 0 | 0 | 1,370 | 97 | 0 | 0 |
|  | Small Town/Rural | 0 | 0 | 0 | 0 | 2,232 | 100 | 0 | 0 | 2,232 | 100 |
| Sources: U.S. Department of Agriculture - Economic Research Services, Frontier and Remote (FAR) Area Codes, 2010, Census Tract-level U.S. Department of Agriculture - Economic Research Services, Rural-Urban Commuting Area (RUCA) Codes, 2010, Census Tract-level  Notes:  ^a^Metropolitan (RUCA 1-3); micropolitan (RUCA 4-6) and small town/rural: (RUCA 7-10). ^b^Frontier-metropolitan tracts were reclassified as frontier-micropolitan.  ^c^Percentages do not add up to 100% due to the nested nature of the FAR codes. | | | | | | | | | | | |

## **A2. Figures**

| Figure S1. Sensitivity analysis of median distance (in miles) to various health facilities in the West Region including all states, compared with the West Region excluding Alaska, 2010 |
| --- |
| 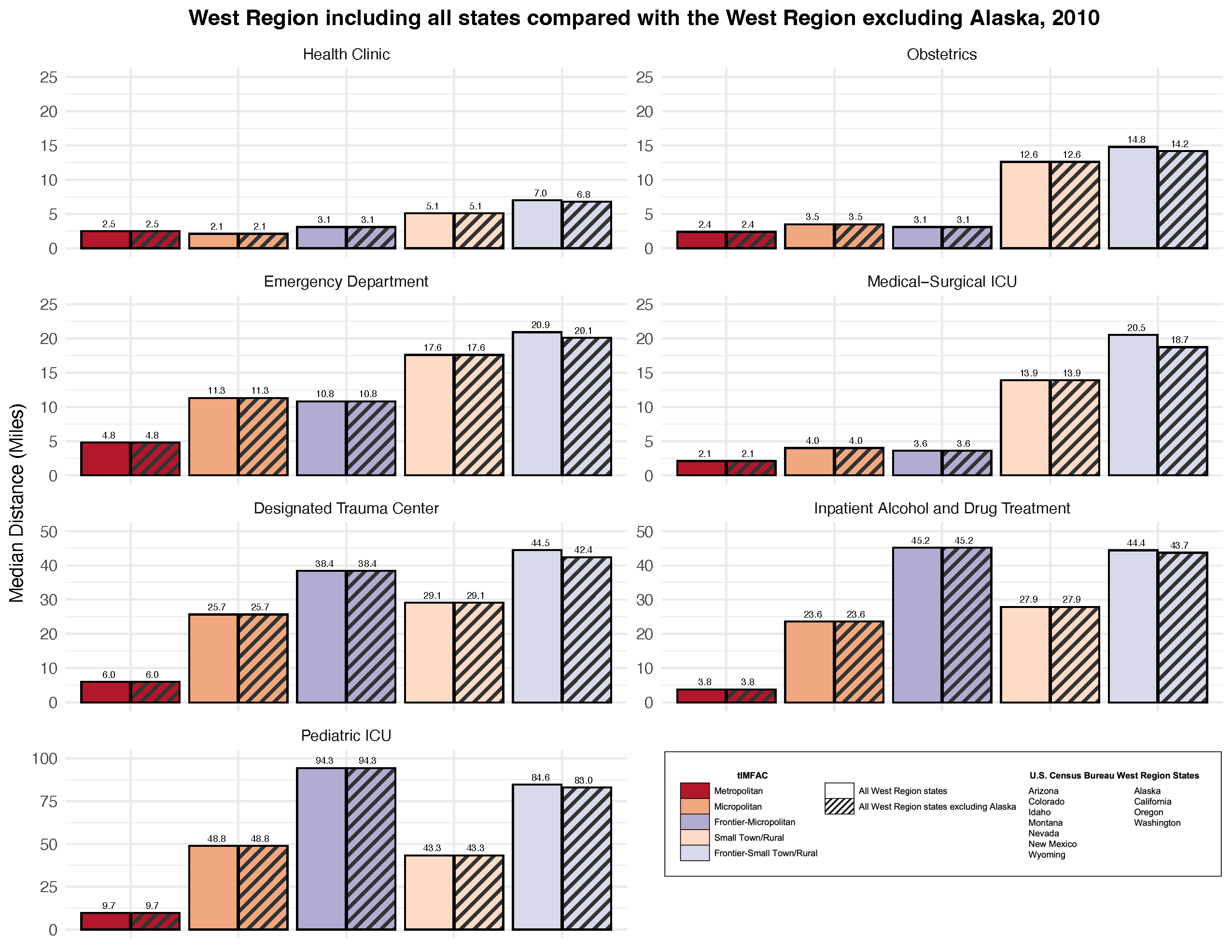 |
| Source: AHRQ Social Determinants of Health Database, 2010 Census Tract Data |

| Figure S2. Sensitivity analysis of median distance (in miles) to various health facilities in the US Census Bureau-defined West Region including all states, compared with the West Region excluding Montana, 2010 |
| --- |
| 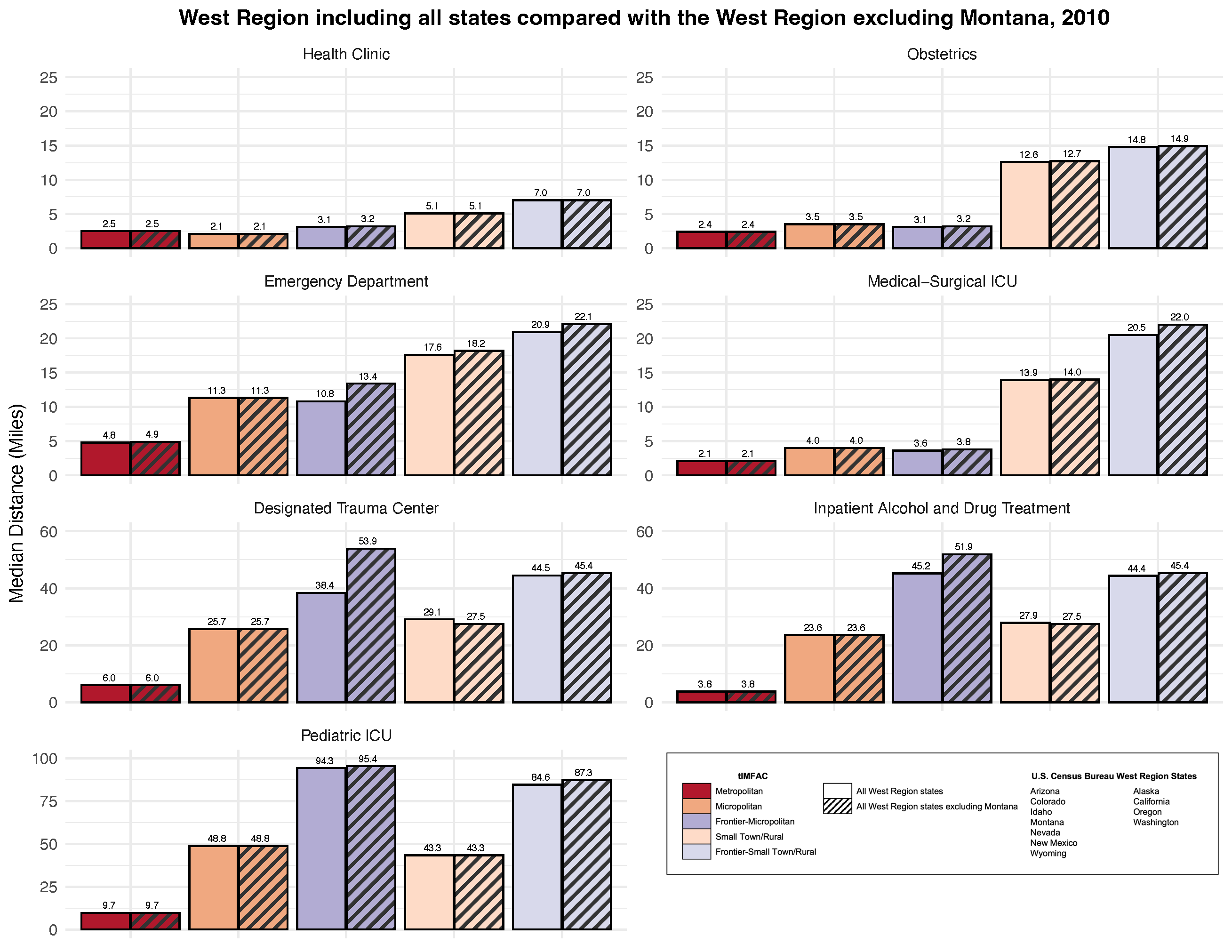 |
| Source: AHRQ Social Determinants of Health Database, 2010 Census Tract Data |

| Figure S3. Sensitivity analysis of median distance (in miles) to various health facilities in the US Census Bureau-defined South Region including all states, compared with the South Region excluding Texas, 2010 |
| --- |
| **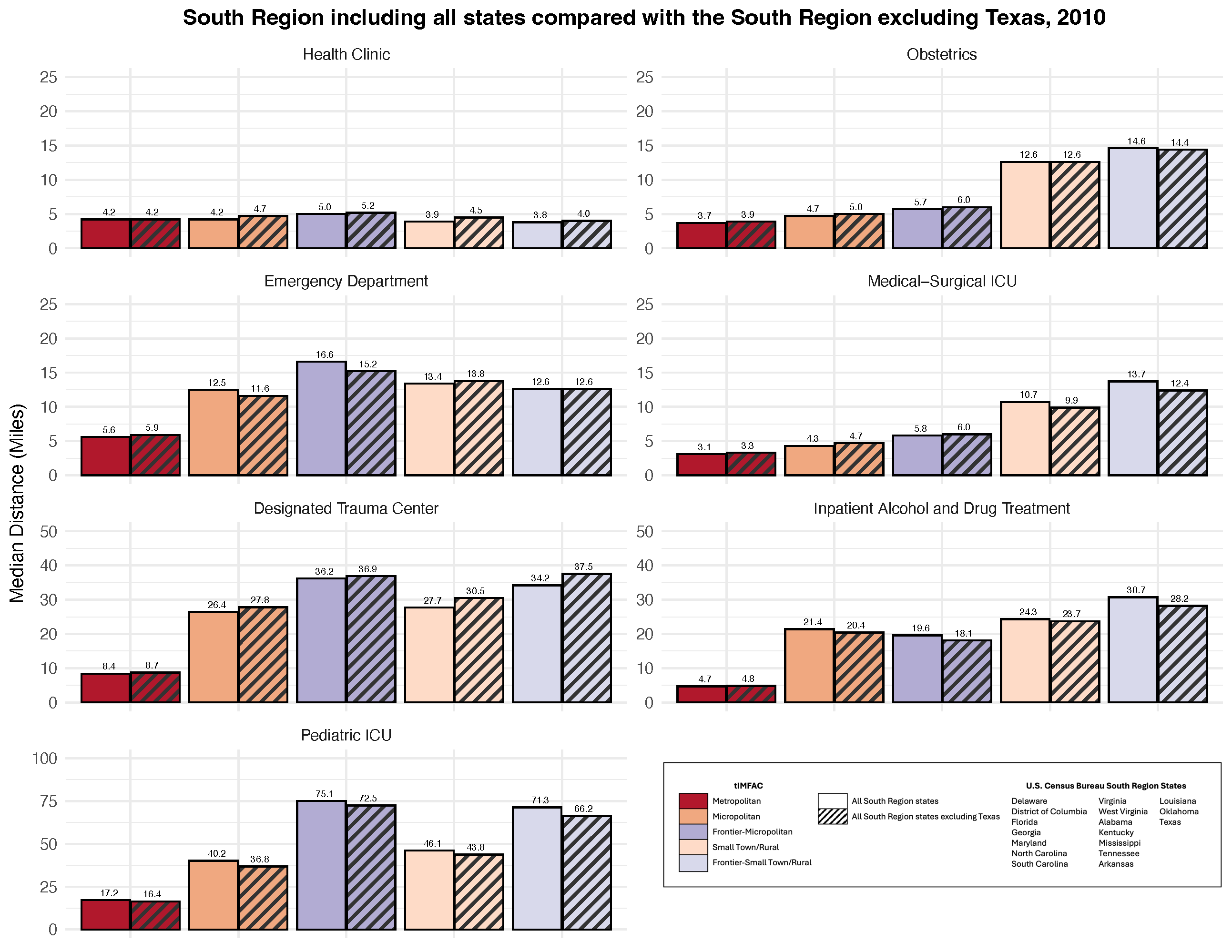** |
| Source: AHRQ Social Determinants of Health Database, 2010 Census Tract Data |

# **Section B. County-level Metropolitan-to-Frontier Area Codes (cIMFAC)**

## **B1. Methods**

| Figure B1**.** Diagram illustrating the combined classification of U.S. counties using the Office of Management and Budget’s (OMB’s) county delineation standards for metropolitan and micropolitan statistical areas and the frontier index score to develop the county-level Integrated Metropolitan-to-Frontier Area Codes (cIMFAC) scheme to assign counties as metropolitan, micropolitan, noncore, frontier-micropolitan, and frontier-noncore |
| --- |
| **** |
| Note: The first step in developing the county-level IMFAC (cIMFAC) was to create a county-level measure of frontier. Since census tracts are nested within counties, we used the tract-based FAR level 1 codes to develop a county-level frontier index score. The frontier index score is the proportion of the county’s population living in tracts classified as FAR level 1, with values ranging from 0 to 1. Counties with frontier index scores of ≥0.5 were classified as frontier, and those with frontier index scores <0.5 were designated as non-frontier. This is consistent with the 50% population threshold that the USDA-ERS used to aggregate grid-level frontier area measures to larger geographic units (i.e., census tracts and zip codes). Frontier areas classified using this county-level index resulted in a similar population proportion as that defined using the tract-level FAR 1 code (3.8% vs. 4.0%, respectively). Next, we cross-classified county-level OMB metropolitan and micropolitan statistical area categories and county frontier status. This resulted in six categories: metropolitan, micropolitan, noncore, frontier-metropolitan, frontier-micropolitan, and frontier-noncore. The frontier-metropolitan group contained two counties (n = 2,981 people), which were reclassified as frontier-micropolitan, aligning with the same logic used for the tract-level IMFAC (tIMFAC) classification. |

## **B2. Summary Tables**

| Table B1. U.S. population and land area classified according to county-level OMB, Frontier Index Score, and cIMFAC geographic schemes, 2010 | | | | | | | |
| --- | --- | --- | --- | --- | --- | --- | --- |
| ***Scheme*** | ***Classification*** | ***Population*** *(n=*308,745,538*)* | ***Land Area*** *(m^2^)* | ***Population Density*** *(persons/m^2^)* | ***Counties*** | ***% Population*** | ***% Land Area*** |
| **OMB^a^** | **Metropolitan** | 262,452,132 | 978,331 | 268 | 1,167 | 85 | 28 |
|  | **Micropolitan** | 27,154,213 | 695,792 | 39 | 641 | 8.8 | 20 |
|  | **Noncore** | 19,139,193 | 1,857,806 | 10 | 1,335 | 6.2 | 53 |
| **Frontier Index Score^b^** | **Frontier** | 11,601,490 | 1,637,922 | 7 | 768 | 3.8 | 46 |
| **cIMFAC** | **Metropolitan** | 262,449,151 | 976,087 | 269 | 1,165 | 85 | 28 |
|  | **Micropolitan** | 20,935,730 | 393,651 | 53 | 452 | 6.8 | 11 |
|  | **Noncore** | 13,759,167 | 524,269 | 26 | 758 | 4.5 | 15 |
|  | **Frontier-Micropolitan** | 6,221,464 | 304,384 | 20 | 191 | 2.0 | 8.6 |
|  | **Frontier-Noncore** | 5,380,026 | 1,333,537 | 4 | 577 | 1.7 | 38 |
|  | ***(Frontier total)^c^*** | *(11,601,490)* | *(1,637,922)* | *(7)* | *(768)* | *(3.8)* | *(46)* |
| Sources:  U.S. Census Bureau, Census of Population and Housing, 2010. Summary File 1 U.S. Census Bureau. Tiger/Line Shapefiles, 2010  Notes:  ^a^ The U.S. Office of Management and Budget's standard for delineating metropolitan and micropolitan statistical areas at the county level ^b^ The frontier index score represents the proportion of the county's population residing in tracts classified as FAR level 1, with values ranging from 0 to 1. Counties with scores of 0.5 or greater were designated as frontier, while those below 0.5 were classified as non-frontier.  ^c^ cIMFAC frontier total includes frontier-micropolitan and frontier-noncore combined. These two IMFAC frontier categories combined comprise all counties defined by the frontier index score as frontier.  Abbreviations: OMB: Office of Management and Budget  cIMFAC: County-level Integrated Metropolitan-to-Frontier Area Codes  m^2^: square miles | | | | | | | |

## **B3. Agreement between cIMFAC and tIMFAC classifications**

| Table B2. Agreement between the county-level IMFAC (cIMFAC) and tract-level IMFAC (tIMFAC) classifications of the U.S. population, 2010 | | | | | | | | | | | |
| --- | --- | --- | --- | --- | --- | --- | --- | --- | --- | --- | --- |
| **cIMFAC** | **tIMFAC** | | | | | | | | | | **Total** |
|  | **Metropolitan** | | **Micropolitan** | | **Small Town/Rural** | | **Frontier-Micropolitan** | | **Frontier-Small Town/Rural** | |  |
|  | **n** | **%** | **n** | **%** | **n** | **%** | **n** | **%** | **n** | **%** |  |
| **Metropolitan** | 252,295,830 | 96 | 5,841,912 | 2.2 | 4,023,326 | 1.5 | 112,410 | 0.0 | 175,673 | 0.1 | 262,449,151 |
| **Micropolitan** | 2,472,261 | 12 | 15,425,368 | 74 | 2,384,189 | 11 | 416,528 | 2.0 | 237,384 | 1.1 | 20,935,730 |
| **Noncore** | 2,924,840 | 21 | 714,189 | 5.2 | 9,474,422 | 69 | 94,295 | 0.7 | 551,421 | 4.0 | 13,759,167 |
| **Frontier-Micropolitan** | 6,819 | 0.1 | 275,387 | 4.4 | 137,899 | 2.2 | 4,695,915 | 75 | 1,105,444 | 18 | 6,221,464 |
| **Frontier-Noncore** | 15,487 | 0.3 | 31,496 | 0.6 | 279,087 | 5.2 | 276,466 | 5.1 | 4,777,490 | 89 | 5,380,026 |
|  |  |  |  |  |  |  |  |  |  |  |  |
| **Total** | 257,715,237 | 83 | 22,288,352 | 7.2 | 16,298,923 | 5.3 | 5,595,614 | 1.8 | 6,847,412 | 2.2 | 308,745,538 |
|  |  |  |  |  |  |  |  |  |  |  |  |
| **Cohen's Kappa** | 0.75 |  |  |  |  |  |  |  |  |  |  |
| Source:  U.S. Census Bureau, Census of Population and Housing, 2010. Summary File 1 U.S. Census Bureau. Tiger/Line Shapefiles, Census Tracts and Urban Areas, 2010: https://www.census.gov/programs-surveys/geography.html  Notes: The agreement between cIMFAC and tIMFAC in the distribution of the 2010 U.S. population was assessed using Cohen's kappa coefficient, which showed substantial agreement between the classifications (k = 0.75).  Abbreviations:  cIMFAC: County-level Integrated Metropolitan-to-Frontier Area Codes tIMFAC: Tract-level Integrated Metropolitan-to-Frontier Area Codes | | | | | | | | | | | |

## **B4. Maps**

| Figure B2. Maps of the county-level (A) Office of Management and Budget Delineation Standards, (B) Frontier Index Score, and (C) Integrated Metropolitan-to-Frontier Area Codes (cIMFAC) |
| --- |
| 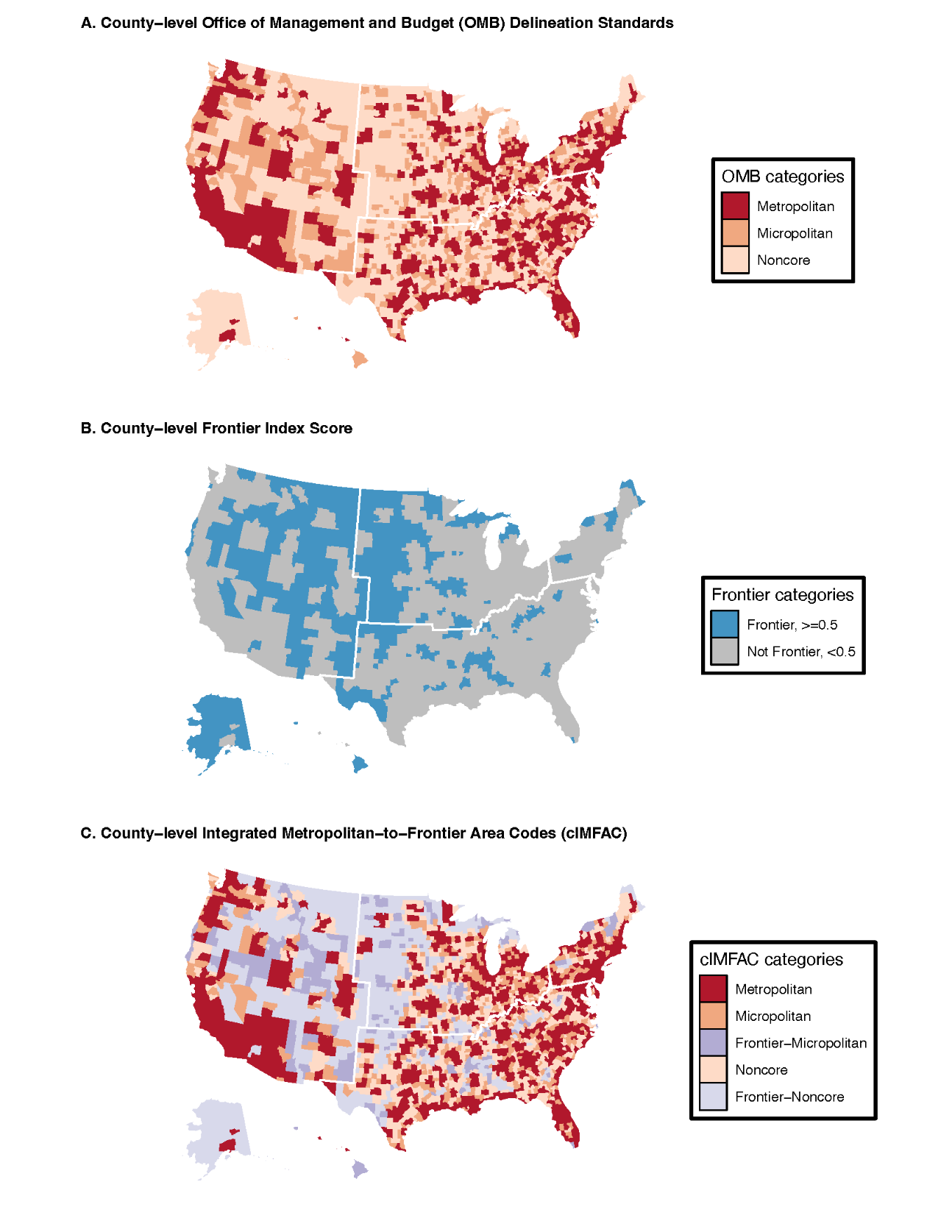 |
| Notes: White borders indicate US Census Region borders. Regions are defined as follows: **Northeast** (Connecticut, Maine, Massachusetts, New Hampshire, Rhode Island, Vermont, New Jersey, New York, Pennsylvania); **Midwest** (Illinois, Indiana, Michigan, Ohio, Wisconsin, Iowa, Kansas, Minnesota, Missouri, Nebraska, North Dakota, South Dakota); **South** (Delaware, District of Columbia, Florida, Georgia, Maryland, North Carolina, South Carolina, Virginia, West Virginia, Alabama, Kentucky, Mississippi, Tennessee, Arkansas, Louisiana, Oklahoma, Texas); and **West** (Arizona, Colorado, Idaho, Montana, Nevada, New Mexico, Utah, Wyoming, Alaska, California, Hawaii, Oregon, Washington). All maps were created in R using the ‘ggplot2’ package. |
